# SHIPi improves hematologic recovery after chemotherapy

**DOI:** 10.1186/s10020-025-01383-3

**Published:** 2025-10-21

**Authors:** Sandra Fernandes, Chiara Pedicone, Otto M. Dungan, Angela Pacherille, Shea T. Meyer, Shawn Dormann, Carlos del Fresno, David Sancho, Bonnie Toms, Brian Walker, Denzil Bernard, John D. Chisholm, William G. Kerr

**Affiliations:** 1 Department of Microbiology & Immunology, SUNY Upstate Medical University, Syracuse, NY USA; 2 Department of Chemistry, Syracuse University, Syracuse, NY USA; 3 Immunomodulation Lab, Innate Immunity Group, IdiPAZ, La Paz University Hospital, Madrid, Spain; 4 Immunobiology Lab, Centro Nacional de Investigaciones Cardiovasculares Carlos III (CNIC), Madrid, Spain; 5 Atomwise, San Francisco, CA USA; 6 Department of Molecular Biology & Biochemistry, SUNY Upstate Medical University, Syracuse, NY USA; 7 Department of Pediatrics, SUNY Upstate Medical University, Syracuse, NY USA

**Keywords:** SHIP1, SHIPi, 5-FU, Chemotherapy, Neutrophils, K161, A32, Candida albicans

## Abstract

The use of recombinant growth factors has led to improved hematopoietic function after chemotherapy, but hematologic and immune complications still occur after chemotherapy which can be life-threatening. Here we show that pan-SHIPi compounds can induce endogenous G-CSF and TPO production in vivo and can also increase host survival after a lethal fungal challenge. In addition, we show that consistent with their ability to induce G-CSF, pan-SHIPi compounds can promote increased granulopoiesis in normal mice and speed recovery of neutrophil counts after chemotherapy. We also report the identification of a novel SHIP1-selective inhibitor, A32, that also increases steady state production of both G-CSF and TPO. These findings indicate small molecule inhibitors of SHIP1 can promote hematologic recovery after myeloablative chemotherapy and are a unique means to induce endogenous production of multiple growth factors that promote hematologic recovery.

**Supplementary Information:**

The online version contains supplementary material available at 10.1186/s10020-025-01383-3.

## Introduction

The majority of cancer patients who receive cytoablative chemotherapies suffer from myelosuppression, including anemia, thrombocytopenia and/or neutropenia (Rostad 1990). The most frequent and deadly complication of myelosuppression is infection related to neutropenia (Rostad 1990; Malech and Gallin 1987). Lineage-specific growth factors such as granulocyte colony stimulating factor (G-CSF), granulocyte–macrophage colony stimulating factor (GM-CSF), erythropoietin (EPO) and thrombopoietin (TPO) have been identified, cloned and clinically developed to address different aspects of myelosuppression (Vadhan-Raj 2003; Vadhan-Raj 2005). However, their efficacy in preventing infectious complications has not been proven (Hubel et al. 2001). In addition, the therapeutic development of TPO for thrombocytopenia has stalled due to immunogenicity of the recombinant formulation that resulted in fatal complications (Vadhan-Raj 2005). The recent development and use of the TPO mimetic, Eltrombopag, in aplastic anemia and immune thrombocytopenia has offered a novel means to promote platelet recovery (Ahmed et al. 2021). More efficacious and cost efficient approaches to enhance blood cell recovery are sought for patients who receive chemotherapy and these include small molecules that target genes which regulate hematopoietic stem/progenitor activity. The SH2-containing 5’-Inositol Phosphatase (SHIP1) is such a molecular target as it limits the bone marrow (BM) niche that supports hematopoietic stem cells (HSC) as well as the in vivo production of both G-CSF and TPO (Hazen et al. 2009; Iyer et al. 2015a; 2015b). We had previously shown that a SHIP1 selective inhibitor 3AC can promote expansion of the HS/PC compartment in vivo and improve blood cell recovery after sublethal radio-ablation. Here we show that second-generation SHIPi compounds with increased potency and solubility can induce both G-CSF and TPO production, enhance blood cell recovery after myeloablative chemotherapy, and improve host survival after a lethal fungal challenge.

**Results**

**Fig. 1:**
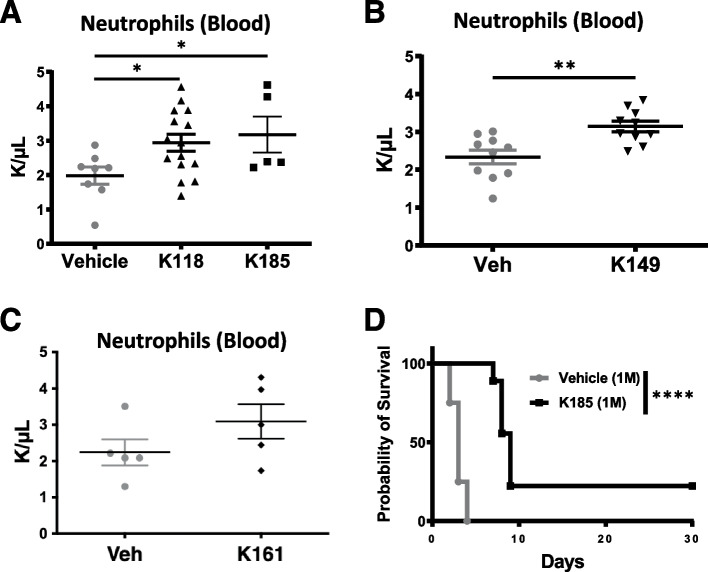
Pan-SHIPi compounds of different chemical classes increase steady-state neutrophil production and function in control of *Candida albicans* infection. **A-C** All compounds were dosed at 10 mg/kg via i.p. injection for 7 days with neutrophils counts in peripheral blood determined on day 8 by automated Hemavet analysis. Each point on the scatterplots in **A**-**C** indicate a value for an individual mouse. **D** A novel SHIP inhibitor K185 protects mice from lethal i.v. *C. albicans* challenge (1 × 10.^6^ cfu). Mice were treated with K185 (10 mg/kg, i.p., *n* = 9) or Vehicle (saline:DMSO, *n* = 8) on days −8, −7 and −1 and on day 0 challenged with 1 million *C. albicans* by i.v. injection. The significance of survival difference between vehicle and K185 dosed mice was determined by a Mantel-Cox log-rank test. The data in all panels are representative of two independent experiments. *p < 0.05, **p < 0.01, ***p < 0.001, ****p < 0.0001

Previously we found that the SHIP1-selective inhibitor 3AC increased neutrophil counts indicating SHIP1 limits steady state granulopoiesis in vivo (Brooks et al. 2010). This phenocopied analysis in SHIP1^−/−^mice as they also exhibit elevated peripheral blood neutrophil counts (Helgason et al. 1998). We then examined whether more potent SHIPi aminosteroids, the stereoisomers K118 and K185, could increase neutrophils counts in mice. K118 and K185 inhibit SHIP1 with greater potency than 3AC, but also significantly inhibit the SHIP1 paralog, SHIP2 (pan-SHIPi), which could compromise the effect of SHIP1 inhibition on granulopoiesis (Pedicone et al. 2020). However, we found that daily administration of K118 or K185 for 7 days increased neutrophil counts significantly vs. vehicle controls (Fig. 1A), indicating that inhibition of SHIP1 while simultaneously inhibiting SHIP2 still leads to an increase in steady state granulopoiesis. We then examined the tryptamine K149, that is also a pan-SHIPi inhibitor (Pedicone et al. 2020), for its capacity to increase peripheral blood neutrophil counts in the steady state. We also found that daily administration of K149 increased blood neutrophil numbers (Fig. 1B). However, not all aminosteroids showed the same potency in increasing neutrophil blood numbers, as the water-soluble pan-SHIPi compound K161 did not increase neutrophil numbers significantly in healthy mice (*p* = 0.09) (Fig. 1C). Neutrophils play an essential role in fending off fungal infections (Desai and Lionakis 2018), and thus increased granulopoiesis promoted by some pan-SHIPi compounds would be expected to increase host resistance to a fungal challenge. Thus, we tested the pan-SHIPi compound, K185, for its ability to increase host resistance to a fungal challenge. (Fig. 1D) K185 and K118 are stereoisomers with essentially equivalent potency for both SHIP1 and SHIP2, but are more potent than K149 (Pedicone et al. 2020). We found that prophylactic dosing of mice with K185 prior to *C. albicans* infection significantly increased survival of C57BL/6 hosts that received a lethal intravenous dose of *C. albicans* (1 × 10^6^ cfu) (Fig. 1D). As SHIP1 has been shown in a genetic model (Helgason et al. 1998) and following treatment with a SHIP1 selective inhibitor 3AC (Brooks et al. 2010) to limit steady state granulopoiesis, the above studies with pan-SHIPi compounds indicate that simultaneous co-inhibition of SHIP2 does not compromise the capacity of these compounds to increase neutrophil production and their function in controlling *Candida albicans *infection *in vivo*. These new findings with K185 in a fungal challenge model and the previous protection observed in a Leishmania challenge model observed with 3AC (Chowdhury et al. 2023) suggest SHIP1 inhibitory aminosteroids could have significant therapeutic potential for protection from lethal infection.

In previous studies with 3AC and more potent pan-SHIPi compounds all were delivered via intraperitoneal injection which is not clinically acceptable. We then explored different routes of SHIPi administration in order to determine if there is a clinically acceptable route of administration that could induce G-CSF production. Administration of either 3AC or K118 via oral gavage was not able to significantly induce G-CSF, although mice dosed in parallel via i.p. route did show induction of G-CSF production (Suppl. Figure 1 A). The same trend was observed with K185 as oral gavage did not significantly increase serum G-CSF in mice, but i.p. administration did (Suppl. Figure 1B). We then explored subcutaneous (subQ) administration with the pan-SHIPi compound K149 and compared this route in parallel to i.p. administration and found that subQ administration of K149 potently increased G-CSF production at 10 mg/kg, 30 mg/kg and 100 mg/kg (*p* = 0.06) (Suppl. Figure 1 C). We then confirmed that subQ administration of K149 induced G-CSF, but compared it in parallel to subQ administration of K161. SubQ administration of either the tryptamine K149 or the aminosteroid K161 was able to potently induce G-CSF and particularly at a dose of 30 mg/kg (Suppl. Figure 1D). We also tested the ability of SHIP1-selective inhibitor 3AC to induce TPO following i.p. administration in healthy C57BL/6 mice and found that, like in SHIP1^−/−^mice (Hazen et al. 2009), SHIP1 inhibition via 3AC increased basal TPO production (Suppl. Figure 1E). We then tested whether the water-soluble pan-SHIP1/2 inhibitor K161 could induce TPO in mice that had undergone a myeloablative regimen of 5-fluorouracil (5FU) for 5 days, followed by 3 days of daily subQ K161 administration at 10 or 30 mg/kg. We found that K161 boosted TPO production post-myeloablation at 30 mg/kg (Suppl. Figure 1 F), and also enhanced neutrophil recovery after the 5-FU regimen (Suppl. Figure 1G). Taken together these data show that subQ administration of potent pan-SHIP1/2 inhibitors can induce G-CSF and TPO production, including for the latter in myeloablated hosts.

**Fig. 2:**
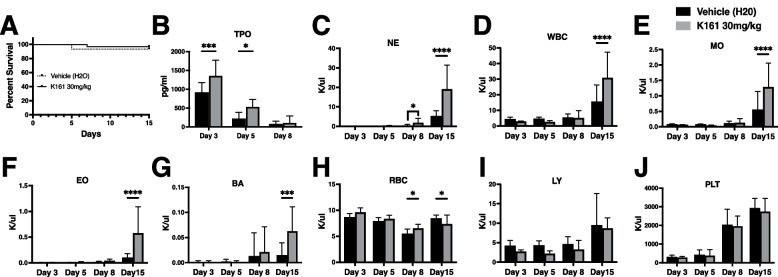
K161 enhances TPO production and blood cell recovery after chemotherapy. Mice were treated daily for 5 days with 5-FU at 35 mg/kg by intraperitoneal injection, followed by 6 daily subcutaneous injections with 30 mg/kg of K161 or vehicle (H_2_O). **A** Survival of the K161 and vehicle (H20) treated mice after a 5 day regimen of 5FU (35 mg/kg).(*p* > 0.05, Mantel-Cox log-rank test) (**B**) Plasma thrombopoietin (TPO) is significantly increased by K161 treatment at 3 and 5 days following 5FU treatment. **C** Neutrophils (NE) production is significantly increased on days 8 and 15 post-5FU with K161 treatment. Production of leukocytes (**D**, WBC), monocytes (**E**, MO), eosinophils (**F**, EO) and basophils (**G**, BA) are increased at 15 days post-5FU in the K161 treated group vs. vehicle controls. **H** Red blood cell (RBC) recovery is also significantly lower at day 8 post-5FU, but reduced at day 15 vs. vehicle controls. No significant effect was observed on lymphocyte (**I**, LY) or platelet counts (**J**, PLT) post-5FU. Significance of the above blood counts was assessed by a two-way ANOVA, *p < 0.05, ***p < 0.001, ****p < 0.0001, *n* = 18/group

The pan-SHIPi compound K161 has greater promise due to its complete water solubility and 4–5 fold higher potency for inhibition of both SHIP1 and SHIP2 as compared to K149 (Pedicone et al. 2020). As K161 is completely water soluble we tested K161 vs. a water-only vehicle cohort. We observed no significant impact on survival in the K161 group vs. the vehicle group (Fig. 2A). We again observed that K161 significantly enhanced TPO production shortly after 5-FU dosing (days 3 and 5, Fig. 2B) and increased both the speed and the degree of neutrophil recovery (days 8 and 11 post-5FU, Fig. 2C). K161 also improved post-5FU recovery of other blood cell components, including (Fig. 2D), monocytes (Fig. 2E), eosinophils (Fig. 2F) and basophils (Fig. 2G). K161 did not have a consistent impact on RBC counts (Fig. 2H). The findings in Fig. 2C-G might indicate hematopoietic output promoted by K161 in a recovery setting is biased toward the myeloid arm of hematopoiesis at the expense of the lymphoid compartment. However, recovery of lymphocytes numbers post-5FU were not significantly reduced by K161 treatment (Fig. 2I). In addition, there was no impact, positive or negative, on platelet recovery (Fig. 2J). These findings indicated that a water-soluble SHIPi compound with low micromolar potency can speed or enhance recovery of multiple blood cell components after myeloablative chemotherapy as well as increase production of a potent growth factors (G-CSF, TPO) that can act on primitive hematopoietic stem/progenitor cells.

**Fig. 3:**
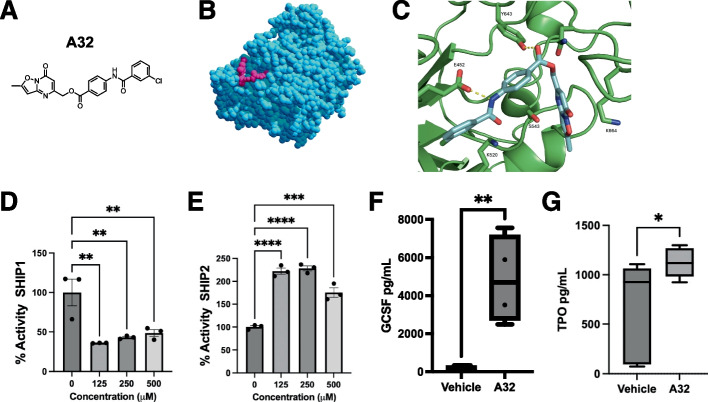
A32 is a potent SHIP1 inhibitor that induces G-CSF and TPO *in vivo*.** A** A32 chemical structure. **B** Computational docking (AutoDock) of A32 with the SHIP1 enzyme domain (PDB:6IBD) (A32 magenta, SHIP1 blue) and (**C**) the active site of SHIP1. Predicted hydrogen bonds of A32 with active site amino acid residues are indicated. Malachite Green enzyme assay data for A32 with (**D**) recombinant SHIP1 enzyme and (**E**) SHIP2 enzyme. The Malachite Green assays in C for SHIP activity are representative of 2–3 independent experiments performed independently by two different researchers. **F** G-CSF and (**G**) TPO are increased in plasma of A32 treated mice one day after IP administration of A32 (30 mg/Kg) as compared to vehicle-treated (DMSO) control mice. The data in (**F**) is representative of two independent experiments with 4–5 mice/group while the study in (**G**) was performed once with 5 mice/group. The AI screen that initially identified A32 as binding to the SHIP1 active site is based on neural net algorithm and screening approach described in (Atomwise 2024). Subsequent computational docking of A32 to the SHIP1 enzyme domain and active site was done using Autodock. *p < 0.05, **p < 0.01, ***p < 0.001, ****p < 0.0001

Employing an artificial intelligence-based computational screening approach (Atomwise 2024) we recently discovered a potent SHIP1 agonist, K306 (Pedicone 2022), with a novel structure. Using the same computational screening procedure as before (Atomwise 2024) we also identified several new SHIP1 inhibitors, including A32 ([1,2]oxazolo[2,3-a]pyrimidin-7-one) (Fig. 3A), which appears to be a new structural phenotype of inhibitor. In silico docking (Bugnon et al. 2024; Rohrig et al. 2023) of A32 to the SHIP1 active site (PDB:6IBD) predicts that the molecule fits well into the SHIP1 active site, with a docking score of−6.9 kcal/mol (Fig. 3B). Closer inspection of the docking pose indicates that two hydrogen bonds (between the amide N–H of A32 and E452, and between the ester carbonyl of A32 and Y643 of SHIP1) (Fig. 3C) hold the molecule in the enzyme active site. While the docking pose does not predict any hydrogen bonds to the isoxazolopyrimidinone of A32, S543 rests near the pyrimidinone nitrogen, and K664 rests near the pyrimidinone carbonyl, so with small bond rotations additional interactions with the heterocycle appear likely. As predicted by the computational screen and docking, A32 was an effective inhibitor for SHIP1, but showed no inhibition of SHIP2 at any concentration tested (Fig. 3D, E). To further confirm that A32 could inhibit SHIP1 we then asked if (as observed for the SHIP1 inhibitors 3AC, K149 and K161) if A32 could also induce G-CSF production *in vivo*. Indeed, i.p. administration of A32 at 10 mg/kg led to a greater than 1000-fold induction of steady state G-CSF production indicating that A32 has potent activity against SHIP1 *in vivo* (Fig. 3F). We further tested whether A32 could induce TPO and also found it significantly increased steady state production of TPO (Fig. 3G). All aminosteroid and tryptamine SHIP1 inhibitors we have identified to date harbor varying degrees of SHIP2 inhibition; however, A32 exhibits no SHIP2 inhibition at any concentration tested. Thus, solely inhibiting SHIP1 is sufficient to induce these two potent hematologic growth factors. These A32 findings are consistent with increased steady state production of both G-CSF and TPO in SHIP1 KO mice (Hazen et al. 2009).

**Discussion**

We have shown here that second generation SHIPi compounds which inhibit SHIP1 but which also have comparable potency against its paralog SHIP2 can mediate significant inductions of both G-CSF and TPO. Consistent with the capacity of these two hematopoietic growth factors to stimulate hematopoietic stem/progenitor proliferation and differentiation, we show that both aminosteroid and tryptamine pan-SHIPI compounds can promote neutrophil increases in normal healthy mice. Consistent with this, we find that one such aminosteroid, K185, can protect against a lethal fungal challenge. Finally, we show that a water soluble aminosteroid pan-SHIPi compound K161 can induce TPO production after a myeloablative 5-day regimen of 5FU, a potent chemotherapeutic that has significant hematologic toxicity. Consistent with this, we saw a more rapid recovery of neutrophils, but also improved recovery of other blood cells including monocytes and white blood cells. These findings show that more potent second-generation SHIP inhibitors that inhibit SHIP1, but which also inhibit SHIP2, are still able to promote blood cell recovery and importantly in the setting of recovery from myeloablative chemotherapy. These improved SHIPi compounds and new ones that are being identified by artificial intelligence guided approaches (Atomwise 2024) like the isoxazolopyrimidinone A32 might now be explored clinically in the treatment of chemotherapy-related blood cell nadirs, but also possibly in myelodysplastic syndromes and anemias.

## Materials and methods

### Mice

C57BL/6 J mice were purchased from Jackson Laboratories or Taconic and were at least 8 weeks old at the time of experimentation. All mice were housed at the Upstate Medical University Department of Laboratory Animal Resources Facility for at least one week prior to start of experimentation under conventional immunocompetent housing and feeding conditions. All experiments were performed with the approval of the Institutional Animal Care and Use Committee. For the *C. albicans* challenge studies C57BL6/J mice were bred in house at the CNIC Vivarium (Madrid).

### SHIPi dosing of mice

Dosing of mice with SHIPi compounds was by the route indicated (intraperitoneal, oral gavage or subcutaneous) for each study and was performed at 5ul volume/g of body mass at the concentration indicated for each experiment or group of mice. For *in vivo* dosing with A32 ([1,2]oxazolo[2,3-a]pyrimidin-7-one) we purchased the compound from Mcule Inc (Palo Alto, CA) catalog number: ChemDiv MCULE-7923864476.

### Hemavet analysis of blood cell components

Mice were bled via the facial vein under inhalation anesthesia (isoflurane) using a 25-gauge needle. Blood was collected in a 100uL EDTA-Microvette tube (Cat#20.1278.100, Sarstedt). Blood cell components were then quantitated using a Hemavet 950S automated blood cell analyzer (Drew Scientific).

### Candida albicans challenge studies

*Candida albicans* (strain SC5314, kindly provided by Prof. C. Gil, Complutense University, Madrid) was grown on YPD-agar plates (Sigma) at 30ºC for 48 h. An isolated colony was collected and transferred to 5 ml of YPD liquid medium, incubating it overnight at 30ºC under gentle shaking. Grown *C. albicans* yeasts were washed twice in sterile PBS by centrifugation at 1,500 rpm for 10 min at RT, for a final counting in a Neubauer chamber. Mice were intravenously infected through the tail vein with 10^6 *C. albicans* in 200μL sterile PBS, and monitored daily for weight, general health, and survival, following the institutional guidance at CNIC (Madrid).

### 5-FU treatment of mice

Mice were treated daily for 5 consecutive days with 5-FU (F6627, Sigma, 3.5 mg/mL in Sterile PBS) at 35 mg/kg by intraperitoneal injection with a 29-gauge needle at 10uL/g of body weight.

### ELISA measurements of plasma G-CSF and TPO

Mice were bled via the facial vein under inhalation anesthesia (isoflurane) using a 25-gauge needle. Blood was collected into a Microvette 300 Serum CAT tube (Catalog # 20.1308.100, Sarstedt) and the tube spun at 12,000xg for 5 min to collect cell-free plasma. Plasma levels of G-CSF and TPO were then determined by ELISA (R&D Systems) according to the manufacturer's instructions.

### Synthesis of 3AC, K118, K149, K161 and K185

Chemical synthesis and purification procedures for the aminosteroids 3AC, K118, K161 and K185 compounds (Pacherille et al. 2025) and the tryptamine K149 (Fernandes 2022) have been described in detail.

### SHIP1/2 enzyme assays

Recombinant SHIP1 and SHIP2 was expressed in E.coli and purified as described in Pedicone et al. (2022). Aliquots of SHIP1 or SHIP2 enzymes derived from this purification we then stored in glycerol at−80C and were thawed for all measurements of A32 activity on both SHIP1 and SHIP2 using the Malachite Green assay as described in Pedicone et al. (2022).

### Prediction of A32 binding to the SHIP1 enzyme domain and active site

Computational docking of A32 to the SHIP1 active site (PDB:6IBD) was performed using Autodock Vina (Eberhardt 2021) on the Swissdock server (Bugnon 2024).

### Statistics

All statistical analyses were performed using GraphPad Prism 10.

## Supplementary Information

Supplementary Material 1: Suppl. Fig. 1. SHIPi compounds induce production of G-CSF and TPO. (A) C57BL/6 mice were dosed with 3AC or K118 by either the i.p. route in Klucel or via oral gavage (as indicated) and the next day bled for ELISA quantitation of G-CSF in their serum. For i.p. dosing of 3AC was at 26.5mg/kg and K118 at 10mg/kg, for oral gavage 3AC at 40mg/kg and K118 at 25mg/kg. Note that 3AC i.p. values exceeded the maximum level of detection for the ELISA. (B) C57BL/6 mice were dosed with K185 by either the i.p. route in Klucel (20mg/kg) or via oral gavage (100mg/kg) as indicated and the mice bled the next day for ELISA quantitation of G-CSF in their serum. (C) Mice were dosed with K149 subcutaneously at 10, 30 and 100mg/kg or vehicle (0.5% DMSO/PBS). The next day serum was harvested and the plasma concentration of G-CSF determined by ELISA (for subQ comparisons vs. vehicle a one-way ANOVA was used, **p<0.01, ****p<0.0001). (D) C57Bl/6 mice were treated by a single subcutaneous injection of K161 or K149 at 10mg/kg, 30mg/kg or Vehicle (0.5%DMSO:H2O). At 16h post-injection, blood was harvested and plasma G-CSF was measured by ELISA (One-way ANOVA, **p<0.01, ****p<0.0001, n=5/group). (E) Mice were dosed with 3AC (26.5mg/kg) via i.p. injection on two consecutive days and the mice bled the next day for ELISA quantitation of serum TPO concentration. (F) K161 (10 or 30mg/kg) was given subcutaneously for three consecutive days to mice following a 5-day regimen of 5FU (35mg/kg, i.p.) and the mice bled the next day for ELISA quantitation of serum TPO concentration or day 15 post-FU for (G) Hemavet quantitation of neutrophil numbers.

## Data Availability

Raw data for all figures, and statistical analysis of such, can be furnished in Prizm (GraphPad) files upon request. All materials can be provided upon completion a standard academic MTA as required by the State University of New York. Chemical compounds can be provided if they are not already commercially available, but this will be dependent on their availability and costs required for their synthesis.

## References

[CR1] Ahmed HAW, et al. Eltrombopag effectiveness and tolerability in chronic immune thrombocytopenia: a meta-analysis. Clin Appl Thromb Hemost. 2021;27:10760296211005556. 33874785 10.1177/10760296211005555 PMC8060759

[CR2] Atomwise AP. AI is a viable alternative to high throughput screening: a 318-target study. Sci Rep. 2024;14(1):7526. 38565852 10.1038/s41598-024-54655-z PMC10987645

[CR3] Brooks R, et al. SHIP1 inhibition increases immunoregulatory capacity and triggers apoptosis of hematopoietic cancer cells. J Immunol. 2010;184(7):3582–9. 20200281 10.4049/jimmunol.0902844 PMC4123216

[CR4] Bugnon M, et al. Swissdock 2024: major enhancements for small-molecule docking with attracting cavities and autodock vina. Nucleic Acids Res. 2024;52(W1):W324–32. 38686803 10.1093/nar/gkae300 PMC11223881

[CR5] Chowdhury BP, et al. SHIP1 inhibition via 3-alpha-amino-cholestane enhances protection against Leishmania infection. Cytokine. 2023;171:156373. 37776719 10.1016/j.cyto.2023.156373

[CR6] Desai JV, Lionakis MS. The role of neutrophils in host defense against invasive fungal infections. Curr Clin Microbiol Rep. 2018;5(3):181–9. 31552161 10.1007/s40588-018-0098-6 PMC6758935

[CR7] Eberhardt J, et al. AutoDock Vina 1.2.0: new docking methods, expanded force field, and python bindings. J Chem Inf Model. 2021;61(8):3891–8. 34278794 10.1021/acs.jcim.1c00203 PMC10683950

[CR8] Fernandes S, et al. N1-benzyl tryptamine pan-SHIP1/2 inhibitors: synthesis and preliminary biological evaluation as anti-tumor agents. Molecules. 2022. 10.3390/molecules27238451. 36500543 10.3390/molecules27238451 PMC9738565

[CR9] Hazen AL, et al. SHIP is required for a functional hematopoietic stem cell niche. Blood. 2009;113(13):2924–33. 19074735 10.1182/blood-2008-02-138008 PMC2662639

[CR10] Helgason CD, et al. Targeted disruption of SHIP leads to hemopoietic perturbations, lung pathology, and a shortened life span. Genes Dev. 1998;12(11):1610–20. 9620849 10.1101/gad.12.11.1610 PMC316868

[CR11] Hubel K, et al. Current status of granulocyte (neutrophil) transfusion therapy for infectious diseases. J Infect Dis. 2001;183(2):321–8. 11112098 10.1086/317943

[CR12] Iyer, S., R. Brooks, M. Gumbleton, and W. G. Kerr. SHIP1-expressing mesenchymal stem cells regulate hematopoietic stem cell homeostasis and lineage commitment during aging. Stem Cells and Development. 2015a;24:1073–1081. PMID: 24857423 10.1089/scd.2014.0501 PMC4403265 25525673

[CR13] Iyer, S., Brooks, R., Gumbleton, M. and Kerr, W.G. SHIP1-expressing mesenchymal stem cells regulate HSC homeostasis and lineage choice during aging. Stem Cells and Development. 2015b;24:1073-81. PMID: 25525673 10.1089/scd.2014.0501 PMC4403265 25525673

[CR14] Kerr WG. Inhibitor and activator: dual functions for SHIP in immunity and cancer. Ann N Y Acad Sci. 2011;1217:1–17. 21155837 10.1111/j.1749-6632.2010.05869.x PMC4515353

[CR15] Malech HL, Gallin JI. Current concepts: immunology. Neutrophils in human diseases. N Engl J Med. 1987;317(11):687–94. 3041216 10.1056/NEJM198709103171107

[CR16] Pacherille AM, et al. Aminocholestane and aminoandrostane inhibitors of the SH2 domain-containing inositol 5’-phosphatase (SHIP). ChemMedChem. 2025;20(8):e202400597. 39843392 10.1002/cmdc.202400597 PMC12046972

[CR17] Pedicone C, et al. Pan-SHIP1/2 inhibitors promote microglia effector functions essential for CNS homeostasis. J Cell Sci. 2020. 10.1242/jcs.238030. 31780579 10.1242/jcs.238030 PMC10682645

[CR18] Pedicone C, et al. Discovery of a novel SHIP1 agonist that promotes degradation of lipid-laden phagocytic cargo by microglia. iScience. 2022;25(4):104170. 35465359 10.1016/j.isci.2022.104170 PMC9020084

[CR19] Rohrig UF, et al. Attracting cavities 2.0: improving the flexibility and robustness for small-molecule docking. J Chem Inf Model. 2023;63(12):3925–40. 37285197 10.1021/acs.jcim.3c00054 PMC10305763

[CR20] Rostad ME. Management of myelosuppression in the patient with cancer. Oncol Nurs Forum. 1990;17(1 Suppl):4–8. 2105482

[CR21] Vadhan-Raj S. Future directions with hematopoietic growth factors. J Natl Compr Canc Netw. 2003;1(Suppl 1):S96-101. 19795582

[CR22] Vadhan-Raj S, Cohen V, Bueso-Ramos C. Thrombopoietic growth factors and cytokines. Curr Hematol Rep. 2005;4(2):137–44. 15720963

[CR23] Brooks, S. Iyer, H. Akada, S. Neelam, C. M. Russo, J. D. Chisholm, W. G. Kerr. Coordinate expansion of murine hematopoietic and mesenchymal stem cell compartments by SHIPi. Stem Cells. 2015;33:(3):848–58. 10.1002/stem.1902R. 10.1002/stem.1902 25402778

