# Supplementary figures and images for: SHIPi improves hematologic recovery after chemotherapy
Source: Mol Med. 2025 Oct 21;31:314. doi: 10.1186/s10020-025-01383-3 (PMC12538841; doi:10.1186/s10020-025-01383-3)

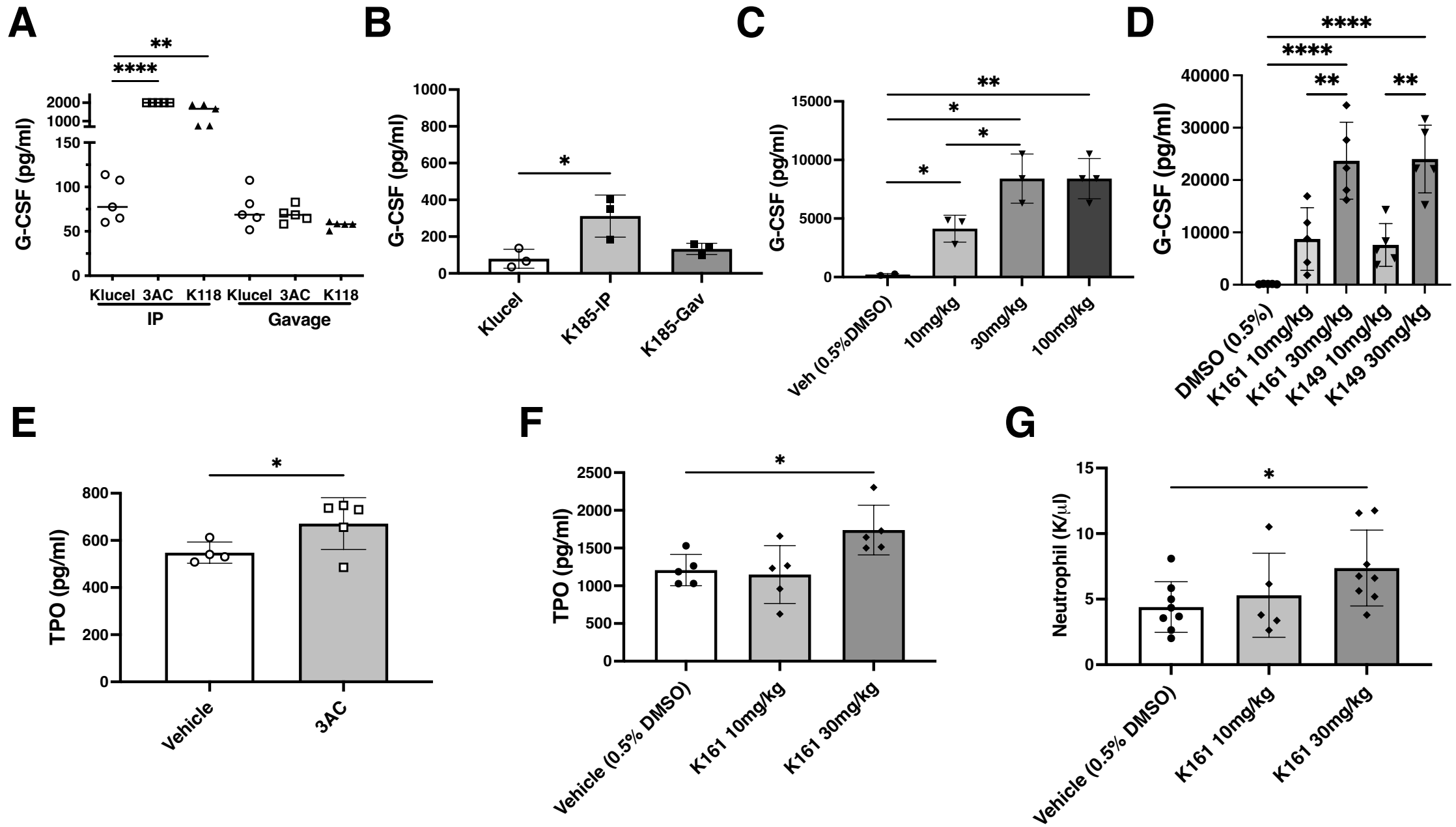

**Suppl. Fig. 1**

Supplement: Supplementary file 1 — Supplementary Material 1: Suppl. Fig. 1. SHIPi compounds induce production of G-CSF and TPO. (A) C57BL/6 mice were dosed with 3AC or K118 by either the i.p. route in Klucel or via oral gavage (as indicated) and the next day bled for ELISA quantitation of G-CSF in their serum. For i.p. dosing of 3AC was at 26.5mg/kg and K118 at 10mg/kg, for oral gavage 3AC at 40mg/kg and K118 at 25mg/kg. Note that 3AC i.p. values exceeded the maximum level of detection for the ELISA. (B) C57BL/6 mice were dosed with K185 by either the i.p. route in Klucel (20mg/kg) or via oral gavage (100mg/kg) as indicated and the mice bled the next day for ELISA quantitation of G-CSF in their serum. (C) Mice were dosed with K149 subcutaneously at 10, 30 and 100mg/kg or vehicle (0.5% DMSO/PBS). The next day serum was harvested and the plasma concentration of G-CSF determined by ELISA (for subQ comparisons vs. vehicle a one-way ANOVA was used, **p<0.01, ****p<0.0001). (D) C57Bl/6 mice were treated by a single subcutaneous injection of K161 or K149 at 10mg/kg, 30mg/kg or Vehicle (0.5%DMSO:H2O). At 16h post-injection, blood was harvested and plasma G-CSF was measured by ELISA (One-way ANOVA, **p<0.01, ****p<0.0001, n=5/group). (E) Mice were dosed with 3AC (26.5mg/kg) via i.p. injection on two consecutive days and the mice bled the next day for ELISA quantitation of serum TPO concentration. (F) K161 (10 or 30mg/kg) was given subcutaneously for three consecutive days to mice following a 5-day regimen of 5FU (35mg/kg, i.p.) and the mice bled the next day for ELISA quantitation of serum TPO concentration or day 15 post-FU for (G) Hemavet quantitation of neutrophil numbers. [file 10020_2025_1383_MOESM1_ESM.pdf]
